# Predictors of New-Onset Diabetes in Hospitalized Patients with SARS-CoV-2 Infection

**DOI:** 10.3390/ijerph192013230

**Published:** 2022-10-14

**Authors:** Iulia Făgărășan, Adriana Rusu, Maria Cristea, Cornelia-Gabriela Bala, Damiana-Maria Vulturar, Ciprian Cristea, Doina-Adina Todea

**Affiliations:** 1 Department of Pneumology, “Iuliu Hațieganu” University of Medicine and Pharmacy, 400332 Cluj-Napoca, Romania; 2 Department of Diabetes and Nutrition Diseases, “Iuliu Hațieganu” University of Medicine and Pharmacy, 400006 Cluj-Napoca, Romania; 3 Faculty of Electrical Engineering, Technical University of Cluj-Napoca, 26–28 G. Barițiu Street, 400027 Cluj-Napoca, Romania

**Keywords:** COVID-19, new-onset diabetes mellitus, predictors, cut-off value

## Abstract

The pandemic caused by the severe acute respiratory syndrome coronavirus 2 (SARS-CoV-2) infection is one of the world’s most disruptive health crises. The presence of diabetes plays an important role in the severity of the infection, and a rise in newly diagnosed diabetes cases has been identified. The aim of this retrospective study was to determine the incidence of new-onset diabetes (NOD) and predictive factors with their cut-off values for patients hospitalized with COVID-19. All patients (*n* = 219) hospitalized for COVID-19 during three consecutive months were included. NOD was diagnosed in 26.48% of patients. The severity of the infection, hospital admission values for fasting plasma glucose, lactate dehydrogenase (LDH), PaO2/FiO2 ratio, the peak values for leucocytes, neutrophils, C-reactive protein, triglycerides, and the need for care in the intensive care unit were predictors for the occurrence of NOD in univariate analysis, while only LDH level remained a significant predictor in the multivariable analysis. In conclusion, the results of the study showed a high incidence of NOD in patients hospitalized with COVID-19 and identified LDH levels at hospital admission as a significant predictor of NOD during SARS-CoV-2 infection. However, the persistence of NOD after the COVID-19 infection is not known, therefore, the results must be interpreted with caution.

## 1. Introduction

The coronavirus disease 19 (COVID-19) pandemic given by the severe acute respiratory syndrome coronavirus type 2 (SARS-CoV-2) is still a public health issue with global impact [1], with a rapid grown worldwide. The new virus responsible for this epidemic wrote a new tab for disasters in human history. Although specific measures for its prophylaxis and treatment—specific vaccines, monoclonal antibody, and antiviral therapy—appear to reduce its morbidity and mortality [2,3], the need for hospitalization and medical care is still high.

It is established that a severe/critical form of COVID-19 increases the risk of a poor prognosis, including death of patients. A number of studies have identified that people who develop those forms of SARS-CoV-2 infection have underlying conditions, such as hypertension (49.7%), obesity (48.3%), chronic lung disease (34.6%), and cardiovascular diseases (27.8%) [4,5,6]. In the studies carried out so far, the morbidity [4] and the mortality [7] in patients with type 2 diabetes mellitus (DM) and the risk for severe/critical forms of COVID-19 were higher than in those without diabetes. This suggests that also DM is a negative prognostic factor [8]. 

It is well-known that DM plays a key role in global morbidity and mortality [9]. Worldwide in 2019, before the outbreak of the SARS-CoV-2 infection, nearly 463 million people aged between 20 and 79 had diabetes as comorbidity [10]. This number it is expected to exceed 700 million by 2045 [10]. Furthermore, it seems that coronavirus is going to alter the International Diabetes Federation (IDF) predictions, given the increased number of hyperglycemia and new-onset diabetes (NOD) diagnosed during SARS-CoV-2 infection and post-COVID-19 [7,11]. 

Thus, a bidirectional link has emerged between DM and SARS-CoV-2 infection: having diabetes increases the risk of developing a severe form of the disease and the infection may increase the risk of NOD [12]. A variety of explanations for the increased serum glucose levels in patients with coronavirus disease have been proposed, including a downregulation of angiotensin-converting enzyme 2 (ACE-2) receptors leading to angiotensin II overproduction [7]. From a physio-pathological point of view, the SARS-CoV-2 virus binds to ACE-2 receptors which are found in kidneys, small intestine, adipose tissue, and in pancreatic beta cells [13]. Therefore, it is plausible to cause a pleiotropic disruption of the glucose metabolism by increasing the production of angiotensin II, leading to a reduction of insulin secretion with increased hepatic glucose production and widening tissue resistance to the action of insulin released into the circulation [7,14]. Other proposed mechanisms for hyperglycemia observed during and after SARS-CoV-2 infection is the direct damage of pancreatic beta cells by the virus itself, as well as the effect of oxidative stress on hypoxia and inflammation [7]. 

To date, as far as we know, there are no previous data regarding the bio-humoral markers to predict the development of diabetes in SARS-CoV-2 infection. The aims of this study were to describe the incidence of NOD in patients with SARS-CoV-2 infection and to investigate these biological predictors of NOD and their cut-off values.

## 2. Materials and Methods

### 2.1. Study Design and Patient Selection

This study was a retrospective study conducted on a consecutive series of patients admitted to the “Leon-Daniello” Pulmonology Hospital in Cluj-Napoca, Romania between 10 November 2020, and 31 January 2021 with a confirmed SARS-CoV-2 infection. 

Patients with all forms of COVID-19 severity were included in the research if their age was above 18 years and the COVID-19 was confirmed through real time-polymerase chain reaction (RT-PCR) according to World Health Organization (WHO) case definition applicable in 2020 [15]. The exclusion criteria were: (1) pregnant women, (2) hospital stay less than 2 days (given that one of the diagnostic criteria used was the presence of 2 basal blood glucose above 126 mg/dL), (3) missing biological samples or imaging investigations. Moreover, given the main objective of this research we excluded from the analysis patients with a known DM, based on medical history or medical files. 

To diagnose NOD during the hospitalization, the American Diabetes Association (ADA) criteria were used: 2 values of fasting plasma glucose (FPG) ≥7.0 mmol/L (≥126 mg/dL) or HbA1c ≥ 6.5% or random glucose level ≥ 11.1 mmol/L (≥200 mg/dL) along with symptoms of hyperglycemia in the absence of a medical history of hyperglycemia [16].

### 2.2. Clinical Data

For each participant, there were registered demographic data: age, gender, body mass index (BMI), medical history (cardiovascular disease, respiratory disease, chronic kidney disease, cancer, and the treatment followed at home), symptomatology (fever, cough, dyspnea, diarrhea, ageusia, other gastro-intestinal troubles, loss of consciousness, pain), and peripheral oxygen saturation (SpO2) at admission. Other data collected included the number of days since COVID-19 symptoms onset until hospitalization, the evolution of the respiratory status with progression to a critical form of COVID-19, with the need for an intensive care unit (ICU). The severity of COVID-19 was determined according to the Romanian Protocol for the therapy of patients with SARS-CoV-2 infection [17] as follows: mild type included patients with clinical symptoms without abnormal radiological findings; moderate forms included patients with pneumonia on chest computed tomography (CT) without fulfilling any criterion for severe disease; severe forms included patients with respiratory distress, a respiratory rate ≥30 per minute, SpO2 ≤ 93%, or partial pressure of arterial oxygen/concentration of oxygen inhaled (PaO2/FiO2 ratio) ≤300 mmHg; the critical form included patients with respiratory failure with the need of mechanical ventilation, sepsis, multisystem organ failure and with the need of ICU care.

### 2.3. Bloodwork Data

For this study, there were collected parameters routinely assessed to detect the predictors of NOD in SARS-CoV-2 infection. Each of the biomarkers is accessible in any medical service: complete blood count, C-reactive protein (CRP), D-Dimer, ferritinemia, interleukin-6, procalcitonin, lactate dehydrogenase (LDH), FPG, triglycerides, creatinine, aspartate aminotransferase (ASAT), alanine aminotransferase (ALAT), and arterial blood gas. All analyses were performed using standard clinical chemistry techniques in the clinical laboratory of the hospital where the study was performed. The samples were collected in the morning before breakfast and we extracted data from the first day of admission, during hospitalization (days 5–7), and upon discharge.

### 2.4. Statistical Method

The software used for statistical data processing were IBM SPSS Statistics V26.0 (Armonk, NY, USA: IBM Corp) and MedCalc 20.026 (Ostend, Belgium: MedCalc Software Ltd.). SPSS was used to find out the correlation between variables and for uni and multivariate logistic regression, while MedCalc was used to identify the cut-off values of continuous variables assessed as risk factors for NOD and ROC, AUC curves. 

Baseline characteristics (age, gender, smoking status, comorbidities, signs and symptoms, time between symptom onset and hospitalization, hospitalization duration) are presented as frequencies and percentages or as median and quartiles 1 and 3 (Q1; Q3). Mann and Whitney and Chi-square tests were used to compare variables between groups with and without NOD. All variables that were statistically significantly different between groups (with a *p*-value < 0.05) were included in the univariate logistic regression. Variables significantly associated with NOD in univariate logistic regression were further included in a multivariable regression analysis. For the variables associated with NOD in the multivariable logistic regression, receiver-operating characteristic (ROC) curve analysis, area under the curve (AUC), sensitivity, and specificity were used to test the predictive power and to determine cut-off values of risk factors for NOD. The optimal cut-off values of continuous variables assessed as risk factors for NOD were identified using the Youden index method [18] which was built-in in MedCalc. By this method the optimal cut-off point is the point maximizing the difference between the true positive rate and false positive rate [19,20]. All tests were two-tailed and a *p*-value < 0.05 was considered as statistically significant.

### 2.5. Ethics Considerations

The study was conducted in accordance with the Declaration of Helsinki and approved by the Ethics Committee of the “Iuliu-Hațieganu” University of Medicine and Pharmacy Cluj-Napoca, Romania (approval No 2/03.01.2022). Given the retrospective nature of the research, we took the information from the database of the hospital, and the signature of informed consent by participants was not required, as per local regulations.

## 3. Results

### 3.1. Characteristics of Enrolled Patients and NOD Incidence

**Figure 1:**
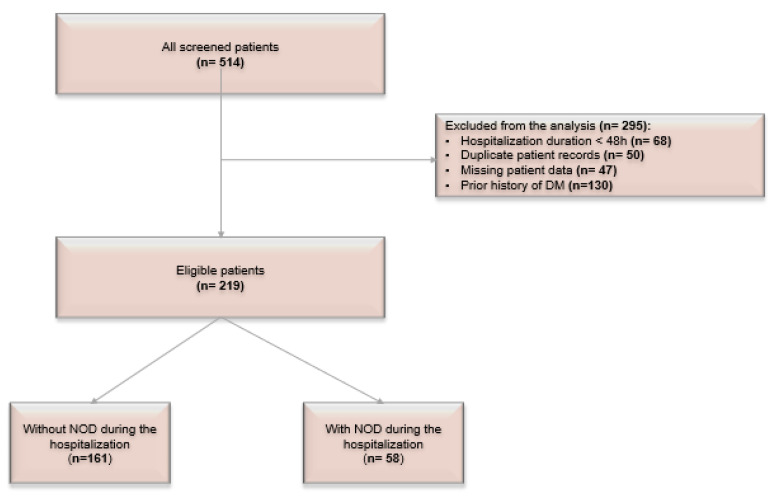
Flow chart of the population. NOD—new-onset diabetes.

Of the 514 patients hospitalized for COVID-19 during the study period, 165 were excluded due to the short duration of hospitalization (<48 h), missing investigations, or readmission of the same patient. Additionally, 130 patients had a prior diagnosis of DM and were excluded from the current analysis. Figure 1 shows the flowchart of the patients’ selection. 

Of the 219 patients who fulfilled the inclusion criteria, had no exclusion criteria, and were included in this analysis, 58 had NOD diagnosed during the hospitalization, giving an incidence of NOD in the present population of 26.5%. 

Table 1 summarizes demographic and clinically relevant data of patients with and without NOD. The median age was 69 years, and the majority were men. Dyspnea and taste loss were the most frequent symptoms encountered in the NOD group than in patients from the control group during SARS-CoV-2 infection (*p* < 0.05). Moreover, patients with NOD more often developed a severe form of COVID-19 compared to those without NOD (*p* = 0.001) and had a more frequent need for ICU care (*p* = 0.043). The median length of hospital stay was also longer (12.5 vs. 9.0 days), with a *p*-value < 0.0001. No statistical significance was observed between the two groups regarding the use of corticoid therapy (*p* = 0.155), nor regarding the time from symptoms onset to hospital admission or confirmation of COVID-19.

The study has also assessed the frequency of NOD according to the need for ICU care and the severity of COVID-19. There was found a higher frequency of NOD in patients who needed ICU care as compared to those without ICU care (43.3% vs. 23.8%, *p* = 0.043). Moreover, the frequency of NOD increased in parallel with the severity of COVID-19 (3.3% in patients with mild form vs. 20.0% in patients with moderate form vs. 33.8% in patients with severe COVID-19, *p* = 0.001).

### 3.2. Hematological and Bio-Humoral Parameters Analysis

Notable contrast appeared after analyzing differences between groups regarding the blood tests collected on the first day of hospitalization and values detected during the admission period, on days 5–7 of the disease (Table 2 and Table 3). 

Ferritin (*p* = 0.015), LDH and FPG (*p* < 0.0001) at admission were significantly higher in NOD group. In terms of blood gas analysis, hypoxemia was more pronounced among patients with NOD (53.12 vs. 63)—but statistically insignificant (*p* > 0.05), while the acute respiratory distress syndrome (*p* = 0.024) was statistically significant in NOD group than in those without NOD. The peak values of biochemical analysis showed that leukocytosis, neutrophilia, ferritin, CRP, LDH, FPG and triglycerides are significantly higher in NOD patients (*p* < 0.05).

### 3.3. Predictors of NOD

In the univariate logistic regression analysis disease severity, FPG and LDH levels at admission, the peak values for leucocytes, neutrophils, CRP, and the need for ICU were statistically significantly associated with NOD during the hospitalization (Table 4). Odds ratio (OR) were 2.740 for disease severity, 2.447 for ICU need, 1.02 for FPG at admission, 1.001 for LDH at admission, 1.077 for peak leucocyte values, and peak neutrophile values, 1.009 for peak CRP, and 1.005 for peak triglycerides. Lower OR were observed for PaO2/FiO2 ratio at admission and peak IL-6 value.

The 10 variables significantly associated with NOD in the univariate analysis were further included in a multivariable regression model. The full regression model was statistically significant in predicting the occurrence of NOD, with a χ^2^ of 20.7 and *p* = 0.023. Of all variables included, only one had a statistically significant contribution to the model: LDH value at admission (OR = 1.009, 95% CI: 1.001; 1.017, *p* = 0.036; Table 5). 

**Figure 2:**
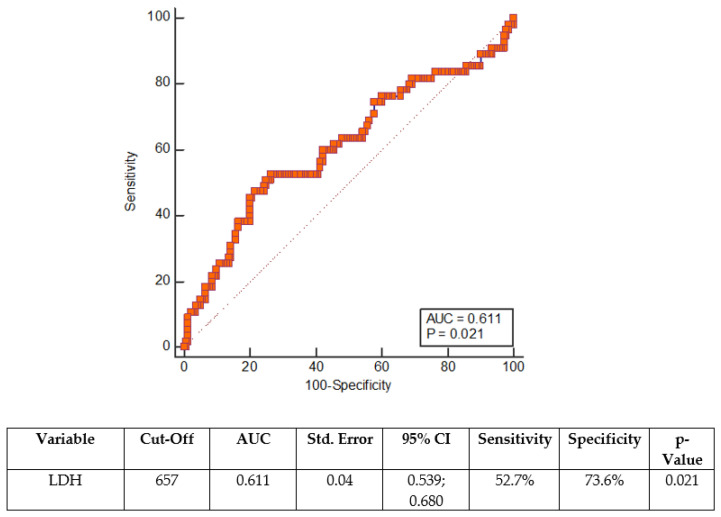
ROC curve for LDH at admission. AUC—area under the ROC curve, ROC—receiver operating characteristics, CI—confidence interval.

The ROC curve was constructed for LDH at admission—Figure 2. 

A cut-off value of 657.0 at hospital admission predicted NOD with a sensitivity of 52.7% and a specificity of 73.6% (Figure 2). 

## 4. Discussion

The present study found a high incidence of NOD in SARS-CoV-2 infection, reaching 26.5%. The incidence was higher in patients who needed ICU care and increased in parallel with the COVID-19 severity. A high incidence of NOD in patients with SARS-CoV-2 infection was also previously reported. In a retrospective study reporting data from 453 patients admitted between January and March 2020 in Wuhan, China, Li et al. [8] reported that 21.0% of COVID-19 patients without a previous diagnosis of DM had an FPG of 7.0 mmol/L or more and/or HbA1c of 6.5% or more. In another study enrolling 184 patients admitted for COVID-19 infection in Livingston, New Jersey between March and May 2020, Smith et al. [21] reported that 15.8% of patients developed type 2 DM during the disease. Moreover, a meta-analysis conducted by Shrestha et al. [12], in which pooled data from six studies were analyzed, showed that 19.7% of patients admitted for SARS-CoV-2 infection were diagnosed with NOD. The incidence of NOD was higher in the present sample compared to other studies, but this could be explained by the higher average age of the patients included in this research than in other previous reports (69.0 years vs. 61.0 [7] or 64.4 years old [21]). Older age is associated with reduced humoral and cell-mediated immune function and age-related comorbidities [22], including DM [23]. The elderly are at a higher risk of developing hyperglycemia due to a combined effect of genetic and lifestyle factors which lead to maladjustment of β-cell secretion to existing insulin resistance [24]. This impaired insulin secretion is due to a natural decline in insulin secretion with age; it has been shown that β-cell secretory capacity is up 50% lower in older adults as compared to young ones [25].

In the multivariable analysis performed, there was found that LDH level at hospital admittance was the only significant predictor of NOD during COVID-19, independent of other parameters. These results are in line with a recent meta-analysis that showed that diabetes is correlated with increased LDH levels in COVID-19 patients [26] as well as with previous studies which showed increased LDH levels in patients with NOD during COVID [7,27]. From a biological point of view, acute lung damage as well as multiorgan damage can be identified by LDH levels. This enzyme is involved in energy production, by turning into lactate to pyruvate. The highest levels are found in the heart, liver, lungs, muscles, kidney, and blood cells [28]. Abnormal values could occur during organ injury or due to decreased oxygenation with up-regulation of the glycolytic pathway [29]. High levels of LDH were correlated with the presence of diabetes; this phenomenon could occur due to several mechanisms: a decrease in glycogen synthesis, a remodel in the oxidative metabolism of glucose, and an increase in glycolytic flux throughout the body [30]. A previous study suggested that high lactate levels in obese patients—where is already a disturbance in glucose transport and metabolism—can significantly influence insulin sensitivity [31]. Moreover, elevated serum LDH concentration could be a predictor of the onset of insulin resistance [32]. These mechanisms are responsible for the increased lactate and insulin resistance in patients with diabetes compared to those without diabetes. This could be explained by more pronounced tissue damage in patients with NOD, as we mentioned before, and by the presence of a more marked inflammatory syndrome, which will be described below [33].

Regarding the COVID-19 severity, the univariate logistic regression showed that patients with NOD had a 2.7 higher risk of a severe form (*p* < 0.001). The association of NOD with COVID-19 severity was previously reported by Birabaharan et al. [34] in patients with SARS-CoV-2 infection. 

The main risk factors for diabetes are overweight and obesity, prediabetes, genetic component, and family history, age, and physical inactivity [35]. Morevoer, a high prevalence of underlying disease was seen in patients with diabetes: hypertension, cardiovascular disease, kidney disease, and stroke [36]. Although in this study the frequency of obesity and associated diseases was slightly higher in the NOD group, it did not reach the statistical significance between groups (*p* > 0.05). Thus, the presence of these comorbidities cannot be associated with the onset of diabetes.

In the present study, the NOD group more often had dyspnea with acute respiratory distress syndrome (ARDS), but with only slightly lower peripheral saturation compared with the control group. This agrees with other studies. Fadini et al. [37] and Farag et al. [27] confirmed that NOD patients had more frequent ARDS than patients without NOD (*p* = 0.001 and *p* < 0.001). The phenomenon was described [38], as “silent hypoxemia”, with the development of thrombi in lung vascular structures, thrombogenesis being often found in patients infected with SARS-CoV-2 [39]. On the other hand, hyperglycemia has a key role in the development of various abnormalities involved in the prothrombotic state encountered in patients with DM [40,41], so this could also explain the increased prevalence of ARDS. 

In the present analysis, higher FPG levels at admission were observed in patients with NOD than in those without. The results are consistent with those of Fadini et al. [37], who found that patients with coronavirus disease and NOD had a markedly increased FPG (89.9 ± 10.3 mg/dl in patients without NOD vs. 208.3 ± 109.9 in patients with NOD, *p* < 0.001). Moreover, the authors found that patients with NOD had a more severe form of SARS-CoV-2 infection, with a higher ARDS and rate of mortality [37]. Similar results were also reported in other papers [42,43]. 

Hyperinflammation and cytokine storm have an important effect on insulin resistance, resulting in hyperglycemia [44]. In the present paper, there was observed a higher peak value for CRP in the NOD group and the univariate analysis suggested that is significantly associated with NOD in coronavirus disease. Likewise, Li et al. [7] and Farag et al. [27], showed higher CRP levels in patients with COVID-19 and DM versus those without DM (18.3 vs. 52.2, *p* < 0.001 and 36.3 vs. 55.4, *p* < 0.009). It is known that patients with diabetes have an immune system imbalance [33] and a higher level of inflammatory biomarkers compared to patients without DM. An important role in this mechanism was attributed to Th17 and Treg cells, along with the secretion of inflammatory factors in patients with DM [45]. The inflammatory process in patients with DM and COVID-19 results from the reduction of the immune response and the increased severity of coronavirus infection in these groups of patients [46].

An extreme form of inflammation is found in patients with a severe form of the coronavirus disease who go through the cytokine storm—an uncontrollable hyperinflammation that can lead to cell damage in multiple locations [44]. During this inflammatory status, an important increase in ferritin was previously observed in patients with NOD versus the control group, with *p* < 0.001 [27]. A retrospective study conducted by Lin et al. [47] showed that the value of ferritin on the first day of hospitalization (OR = 3.302, 95% CI 1.141–9.553, *p* = 0.028) was an independent risk factor for disease severity. Although this study there were also found higher ferritin levels at admission and higher peak values in the NOD group than non-NOD patients (0.015 and <0.001), ferritin was not a predictor for NOD. 

Neutrophils are the main source of cytokines and chemokines. The paradoxical influence of hyperglycemia on neutrophil count is explained by its chemotaxis, reduced phagocytosis produced by neutrophils, macrophages, and monocytes, and lower apoptosis in neutrophils [48,49]. Both diabetes and marked inflammatory syndrome determined by endothelial and lymphocyte cells could increase the neutrophil count leading to an increased SARS-CoV-2 infection severity [49]. In the present study, a higher peak value of leucocytes and neutrophils was associated with NOD. These findings were consistent with prior literature, showing that in coronavirus infection, NOD patients developed higher leukocytosis with neutrophilia than patients without DM [8]. This could be a marker for degradation of the clinical condition, with a high risk of a poor outcome, as shown in a meta-analysis with a total of 3377 COVID-19 patients [50]. The present findings may indicate that patients with DM suffered a more severe viral infection and there was a predilection to bacterial infections; patients coinfected with SARS-CoV-2 and bacterial infection have been studied previously [51]. Moreover, it has been shown that leukocytosis could be an adverse effect of corticosteroids treatment [52], by promoting hepatic gluconeogenesis, reducing the uptake and employment of glucose in peripheral tissues, and increasing the action of other glycemic hormones [53].

Higher hypertriglyceridemia was seen in the NOD group vs. control group (*p* < 0.001). Another recent study showed that, in patients with COVID-19, triglycerides had higher values in the NOD group than in patients without NOD [37]. The mechanism is an increased flow of free fatty acids to the liver which intensifies hepatic triglyceride synthesis [54], suggesting a disorder in lipid and energy metabolism. Both insulin and adiponectin block the disposal of adipocyte-stored fatty acids and thus decrease their plasma level leading to decreased hepatic triglyceride synthesis [55,56]. In COVID-19 patients impaired insulin secretion, insulin resistance and adipose tissue dysfunction occur together [56] and may explain increased free fatty acids levels and triglycerides production [53]. Another potential mechanism involved in the association of triglyceride levels with NOD observed in this study is the use of corticoids, although no difference between groups in the use of corticoid therapy was observed. It is known that glucocorticoids increase insulin resistance in multiple ways: they decrease the number of GLUT 4 muscle transporters, increase the process of lipolysis and proteolysis, stimulate the effects of antagonistic hormones glucagon and epinephrine [57].

The current study showed that in patients with COVID-19, there is a higher percentage of people with NOD who needed ICU than patients without NOD (*p* = 0.043). In 453 patients admitted for SARS-CoV2 infection, NOD patients were more likely to need ICU and invasive mechanical ventilation than normal glucose patients, due to the highest prevalence of coronavirus-related complications and longer hospital stay [8]. Yang et al. [58] concluded that 54% of patients with coronavirus disease and NOD were admitted to ICU.

### Limitations

This investigation has several limitations. The first one is represented by the fact that the persistence of NOD after the COVID-19 infection is not known, therefore, the results must be interpreted with caution. Second, given that it is a retrospective study, certain methodological disadvantages may occur such as limited availability of certain biochemical deteriorations in all patients. Prospective studies are needed to determine the persistence of NOD and the true value of biomarkers analyzed here.

## 5. Conclusions

The present study found a high incidence of NOD in patients hospitalized for SARS-CoV-2 infection and identified LDH levels at hospital admission as a significant predictor of NOD during the SARS-CoV2 infection. LDH could be of real use in closely monitoring the glycemic values with early established antihyperglycemic treatment in order to prevent long-term complications and provide a good quality of life. To the best of our knowledge, this is the first study that highlighted the factors that predict the occurrence of NOD in patients infected with SARS-CoV2. The challenge remains to find the optimal strategy for primary prophylaxis of diabetes and to correct risk factors, regardless of the circumstances in which diabetes may occur. 

## Figures and Tables

**Table 1:** Demographic and clinically relevant data of population.

Characteristics			Total Patients *n* = 219		Without NOD *n* = 161		NOD *n* = 58		*p*-Value
Age, years (median; Q1,Q3)			69.0	[60.0–77.5]	69.0	[60.0–78.0]	66.5	[62.0–74.0]	0.762
Gender, *n* (%)	Men		129	58.9	90	55.9	39	67.2	0.162
Comorbidities, *n* (%)	Obesity (BMI ≥ 30 kg/m^2^)		78	35.6	53	60.2	25	69.4	0.414
	Hypertension		146	66.7	101	62.7	45	77.6	0.051
	Ischemic heart disease		12	5.5	9	5.6	3	5.2	0.247
	Heart failure		31	14.2	24	14.9	7	12.1	0.864
	Atrial fibrillation		27	12.3	21	13.0	6	10.3	0.333
	Stroke		16	7.3	14	8.7	2	3.4	0.109
	Asthma		19	8.7	13	8.1	6	10.3	0.869
	COPD		26	11.9	18	11.2	8	13.8	0.869
	CKD		22	10.0	17	10.6	5	8.6	0.400
	Cancer		25	11.4	20	12.4	5	8.6	0.202
Signs and symptoms, *n* (%)	Fever		97	44.3	69	42.9	28	48.3	0.052
	Cough		148	67.6	105	65.2	43	74.1	0.364
	**Dyspnea**		127	58	85	52.8	42	72.4	**0.013**
	**Taste loss**		14	6.4	13	8.1	1	1.7	**0.047**
	Faintness		6	2.7	6	3.7	0	0.0	0.080
	Pain Chest pain Headache Abdominal pain		23 25 12	10.5 11.4 5.5	14 19 7	8.7 11.8 4.3	9 6 5	15.5 10.3 8.6	0.269
Onset of symptom to, days (median; Q1–Q3)	Hospital admission		3.0	[1.0–7.0]	3.0	[1.0–7.0]	3.5	[2.0–6.5]	0.554
	Confirmation of COVID-19		1.0	[0.0–3.0]	1.0	[0.0–3.0]	1.0	[0.0–3.0]	0.506
**Hospitalization duration, days** **(median; Q1-Q3)**			10.0	[6.0–14.0]	9.0	[5.0–13.0]	12.5	[9.0–16.0]	**<0.0001**
**Disease severity**, *n* (%)		Mild	30	13.7	29	18.0	1	1.7	**0.001**
		Moderate	50	22.8	40	24.8	10	17.2	
		Severe	139	63.5	92	57.1	47	81.0	
Frequency of dexamethasone administration, *n* (%)			174	79.5	123	76.4	51	87.9	0.155
**Need of ICU**, *n* (%)			30	13.7	17	10.6	13	22.4	**0.043**

*n*—number, BMI—body mass index, COPD—chronic obstructive pulmonary disease, CKD—chronic kidney disease, NOD—new onset diabetes, ICU—intensive care unit. *p* values indicate the differences between groups; *p* < 0.05 was considered statistically significant.

**Table 2:** Laboratory findings and arterial blood gas at admission.

Parameters	Total Patients *n* = 219		Without NOD *n* = 161		NOD *n* = 58		*p*-Value
	Median	[Q1; Q3]	Median	[Q1; Q3]	Median	[Q1; Q3]	
White blood cells, ×$10^{3}$/L	7.54	[5.3–10.6]	7.2	[5.2–3.7]	9.4	[5.3–11.2]	0.093
Lymphocyte count, ×$10^{3}$/L	0.96	[0.7–1.4]	1.0	[0.7–1.4]	0.8	[0.6–1.3]	0.195
Neutrophil count, ×$10^{3}$/L	5.81	[3.9–9.1]	5.4	[3.8–8.0]	7.6	[4.1–10.1]	0.051
Hemoglobin, g/dL	13.4	[12.1–14.3]	13.3	[11.8–14.4]	13.5	[12.5–14.3]	0.264
Platelets count, ×$10^{3}$/L	228.5	[177.0–300.0]	230.0	[177.0–302.0]	225.0	[172.0–285.0]	0.603
C-reactive protein, mg/L	54.65	[17.2–89.8]	53.9	[15.7–84.3]	56.4	[25.6–95.2]	0.065
D-dimer, µg/mL	526.0	[259.0–1789.0]	499.5	[175.0–1389.0]	621.0	[352.0–2475.0]	0.224
**Ferritin (ng/mL)**	579.0	[270.9–999.0]	533.1	[259.4–965.0]	769.5	[337.4–1318.5]	**0.015**
**LDH, U/L**	503.0	[387.5–739.5]	488.5	[383.5–664.5]	662.0	[443.5–851.5]	**0.015**
Creatinine (mg/dL)	1.0	[0.9–1.3]	1.0	[0.9–1.2]	1.1	[1.0–1.3]	0.23
Clearance creatinine mL/min/1.73 m^2^	65.0	[51.0–81.0]	67.0	[51.0–82.5]	61.5	[43.0–77.0]	0.416
**FPG, mg/dL**	118.5	[99.0–154.0]	107.5	[95.0–136.0]	149.5	[126.0–166.0]	**<0.0001**
HbA1c, %	5.5	[5.1–6.4]	5.4	[5.4–5.7]	5.5	[5.1–6.5]	1
Triglycerides, mg/dL	149.0	[103.0–202.0]	143.5	[98.0–201.0]	168.0	[117.0–203.0]	0.099
pH	7.5	[7.4–7.5]	7.5	[7.4–7.5]	7.5	[7.4–7.5]	0.621
PaO2, mmHg	58.0	[48.0–73.0]	63.0	[51.0–74.5]	53.1	[47.5–67.5]	0.064
PaCO2 mmHg	34.0	[30.0–38.0]	35.0	[31.0–38.0]	32.0	[28.0–38.0]	0.056
SaO2 %	92.0	[84.0–94.8]	92.5	[87.3–94.6]	86.4	[77.0–95.0]	0.092
HCO3 mmol/L	24.9	[22.5–27.9]	25.2	[23.3–27.9]	23.9	[21.5–26.5]	0.198
**PaO2/FiO2**	122.7	[62.5–211.5]	148.3	[78.9–218.9]	81.6	[51.3–166.2]	**0.024**
A-aDO2	206.6	[117.7–567.3]	172.8	[62.6–551.2]	234.7	[164.7–588.7]	0.061

*n*—number, LDH—lactate dehydrogenase, PaO2—partial pressure of oxygen, PaCO2—partial pressure of carbon dioxide, NOD—new-onset diabetes, FPG—fasting plasma glucose. *p* values indicate the differences between groups; *p* < 0.05 was considered statistically significant.

**Table 3:** Laboratory findings during hospitalization (days 5–7).

Characteristics	Total Patients *n* = 219		Without NOD *n* = 161		NOD *n* = 58		*p*-Value
	Median	[Q1; Q3]	Median	[Q1; Q3]	Median	[Q1; Q3]	
**White blood cells, ×** $10^{3}$ **/L**	10.23	[7.18–13.91]	9.14	[6.78–12.32]	13.85	[10.62–16.81]	**<0.001**
**Neutrophil count, ×** $10^{3}$ **/L**	8.56	[5.3–11.99]	7.19	[4.72–10.85]	10.94	[9.14–14.13]	**<0.001**
Platelets count, ×$10^{3}$/L	298	[221–399]	290.5	[213–387	318.5	[237–431]	0.061
**C-reactive protein, mg/L**	65.7	[21.8–93.8]	59.8	[17.05–92.05]	75.35	[35.5–95.7]	**0.020**
D-dimer, mg/L	858.5	[396–3602]	709	[338–3049]	980	[582–4803]	0.101
**Ferritin, ng/mL**	698	[344.3–1306]	606.6	[259.5–1203]	1012	[596.5–1582]	**<0.001**
Il-6, pg/mL	15.9	[9.49–30.55]	26.5	[13.9–56.49]	15.8	[6.98–25.9]	0.07
**LDH, U/L**	548.5	[399–804.5]	510	[388.5–696]	723	[507–1140]	**0.003**
**AST, U/L**	48	[32–80]	45.5	[30.5–71]	59	[34–99]	**0.017**
**ALT, U/L**	58	[33–103]	55	[29–90]	70	[41–125]	**0.010**
Pro-calcitonin, ng/mL	0.22	[0.18–0.32]	0.21	[0.18–0.31]	0.25	[0.2–0.32]	0.102
**FPG, mg/dL**	138	[105–198.5]	118	[102–158]	223	[185–266]	**<0.001**
**Triglycerides, mg/dL**	192	[140–285]	174	[132–271.5]	257	[191–386]	**<0.001**

LDH—lactate dehydrogenase, AST—aspartate aminotransferase, ALT—alanine aminotransferase, NOD—new- onset diabetes, FPG—fasting plasma glucose. *p* values indicate the differences between groups; *p* < 0.05 was considered statistically significant.

**Table 4:** Univariate logistic regression to find predictors of new-onset diabetes in patients with COVID-19.

	OR (95%CI)	*p*-Value
**Disease severity**	2.740 (1.533; 4.897)	**0.001**
**Need for ICU**	**2.447 (1.104; 5.424)**	**0.028**
Values at hospital admission		
**FPG**	1.020 (1.011; 1.029)	**<0.001**
Ferritin	1.000 (1.000; 1.001)	0.059
**LDH**	1.001 (1.000; 1.002)	**0.024**
**PaO2/FiO2**	0.995 (0.989; 1.000)	**0.047**
Values during hospitalization (days 5–7)		
**Leucocytes**	1.077 (1.029; 1.127)	**0.002**
**Neutrophils**	1.077 (1.026; 1.131)	**0.003**
**CRP**	1.009 (1.00; 1.018)	**0.039**
Ferritin	1.000 (1.000; 1.000)	0.487
**IL-6**	0.980 (0.961; 0.999)	**0.044**
LDH	1.000 (1.000; 1.000)	0.423
AST	1.000 (1.000; 1.001)	0.411
ALT	1.001 (1.000; 1.001)	0.231
**TG**	1.005 (1.002; 1.007)	**<0.001**

FPG—fasting plasma glucose; LDH—lactate dehydrogenase, PaO2—partial pressure of oxygen, FiO2—fraction of inspired oxygen, CRP—C-reactive protein, IL-6—interleukin-6, AST—aspartate aminotransferase, ALT—alanine aminotransferase, TG—triglycerides, ICU—intensive care unit.

**Table 5:** Multivariable regression analysis of risk factors for new-onset diabetes in patients with COVID-19.

	OR (95%CI)	*p*-Value
Disease severity	0.325 (0.036; 2.921)	0.316
Need for ICU	1.906 (0.050; 72.539)	0.728
Values at hospital admission		
FPG	1.016 (0.992; 1.041)	0.188
**LDH**	**1.009 (1.001; 1.017)**	**0.036**
PaO2/FiO2	0.989 (0.968; 1.011)	0.335
Values during hospitalization (days 5–7)		
Leucocytes	1.002 (0.961; 1.044)	0.940
Neutrophils	0.778 (0.585; 1.035)	0.778
CRP	0.984 (0.938; 1.032)	0.501
IL-6	0.889 (0.764; 1.033)	0.125
TG	1.010 (0.995; 1.026)	0.187

ICU—intensive care unit, FPG—fasting plasma glucose, LDH—lactate dehydrogenase, PaO2—partial pressure of oxygen, FiO2—fraction of inspired oxygen, CRP—C-reactive protein, IL-6—interleukin-6, TG—triglycerides.
